# The relationship between prognostic nutritional index and its components (albumin and lymphocyte count) and all-cause mortality in lung cancer patients: a hospital-based study with database external validation

**DOI:** 10.3389/fnut.2025.1649334

**Published:** 2025-09-24

**Authors:** Zhuolin Qin, Longqian Li, Ming Hou, Cheng Wang

**Affiliations:** ^1^The Second Clinical Medical College, Lanzhou University, Lanzhou, China; ^2^Department of Thoracic Surgery, Lanzhou University Second Hospital, Lanzhou, Gansu, China

**Keywords:** PNI, NHANES, cox proportional hazards regression, RCS, VIF

## Abstract

**Objective:**

This study aimed to evaluate the prognostic value of the Prognostic Nutritional Index (PNI), derived from serum albumin and lymphocyte count, in predicting all-cause mortality among lung cancer patients, using both a hospital-based cohort and an external validation dataset.

**Methods:**

A hospital-based retrospective cohort study was conducted, supplemented with external validation using the NHANES database. Univariate and multivariate Cox proportional hazards regression analyses were performed to assess associations between PNI, its components, and mortality. Variance inflation factor (VIF) testing was used to evaluate multicollinearity. Kaplan–Meier (KM) curves and log-rank tests were employed to compare survival across PNI tertiles. Restricted cubic spline (RCS) models were applied to examine non-linear relationships between continuous variables and mortality risk.

**Results:**

In the hospital cohort, univariate Cox analysis revealed significant associations between PNI (HR = 0.89, 95% CI: 0.85–0.93, *p* < 0.01), albumin (HR = 0.88, 95% CI: 0.86–0.92, *p* < 0.01), lymphocyte count (HR = 0.60, 95% CI: 0.50–0.80, *p* < 0.01), and mortality. After multivariate adjustment and VIF testing (all VIF < 5), PNI remained an independent predictor of mortality. KM curves showed significant survival differences across PNI tertiles (log-rank *p* < 0.001). RCS analysis indicated a non-linear relationship between PNI and mortality risk (*p* for nonlinear = 0.007). External validation using NHANES data consistently supported the association between PNI and mortality, with significant survival differences in KM analysis (log-rank *p* = 0.011) and a non-linear trend in RCS.

**Conclusion:**

PNI and its components—albumin and lymphocyte count—are significantly associated with all-cause mortality in lung cancer patients. PNI demonstrates promise as a practical and reproducible prognostic indicator, potentially aiding in risk stratification and clinical decision-making.

## 1 Introduction

Lung cancer continues to be a leading cause of cancer-related deaths globally, with millions of new cases diagnosed annually (1). Despite significant advancements in diagnostics and treatment strategies, including the development of targeted therapies and immunotherapies, the prognosis for lung cancer patients remains grim (2). The complexity of lung cancer biology and its heterogeneous clinical behavior necessitates the identification of robust biomarkers capable of providing accurate prognostic information and guiding personalized treatment approaches (3, 4).

The PNI has gained attention as a composite indicator that encapsulates both nutritional and immune aspects of patient health (5). Derived from serum albumin levels and peripheral blood lymphocyte count, PNI provides a holistic evaluation of an individual’s immune and nutritional status (6). Serum albumin, a major protein constituent of human plasma, serves as a key marker of nutritional status (7). It is intricately involved in maintaining osmotic pressure, transporting various substances, and supporting metabolic functions (8). Low albumin levels are frequently observed in patients with chronic diseases and are associated with increased mortality (9). Lymphocytes, produced by lymphoid organs, are central to immune responses. They participate in both cell-mediated and humoral immunity, helping to identify, target, and eliminate cancer cells. A low lymphocyte count often indicates compromised immune function and is linked to adverse outcomes in various diseases (10, 11).

Since its initial development for assessing preoperative nutritional status and predicting postoperative complications in surgical patients, PNI has been investigated across diverse clinical contexts. Emerging evidence suggests that PNI may serve as a prognostic biomarker in various conditions, including chronic kidney disease, heart failure, and several types of cancer (12–14). For instance, studies have demonstrated that a lower PNI is associated with increased mortality in patients with chronic kidney disease. Similarly, in oncology, PNI has shown promise in predicting outcomes in patients with colorectal cancer, gastric cancer, and hepatocellular carcinoma (15, 16). However, the role of PNI in lung cancer prognosis remains underexplored, with limited studies addressing its potential utility (17, 18).

Given the high mortality associated with lung cancer and the need for effective prognostic markers to guide clinical decision-making, exploring the relationship between PNI and lung cancer outcomes is of significant importance. A better understanding of how PNI and its individual components (albumin and lymphocyte count) influence mortality risk could provide valuable insights for patient care. This hospital-based cohort study, supplemented with external validation using the NHANES database, aims to comprehensively investigate the association between PNI and all-cause mortality in lung cancer patients. By employing a multifaceted analytical approach, including Kaplan–Meier survival analysis, Cox proportional hazards regression, and RCS models, this study seeks to elucidate the complex relationship between PNI and mortality risk. The findings may not only enhance our understanding of the prognostic significance of PNI in lung cancer but also contribute to the development of more personalized and effective management strategies for this deadly disease.

## 2 Methods

### 2.1 Study population and inclusion/exclusion criteria

**Figure 1:**
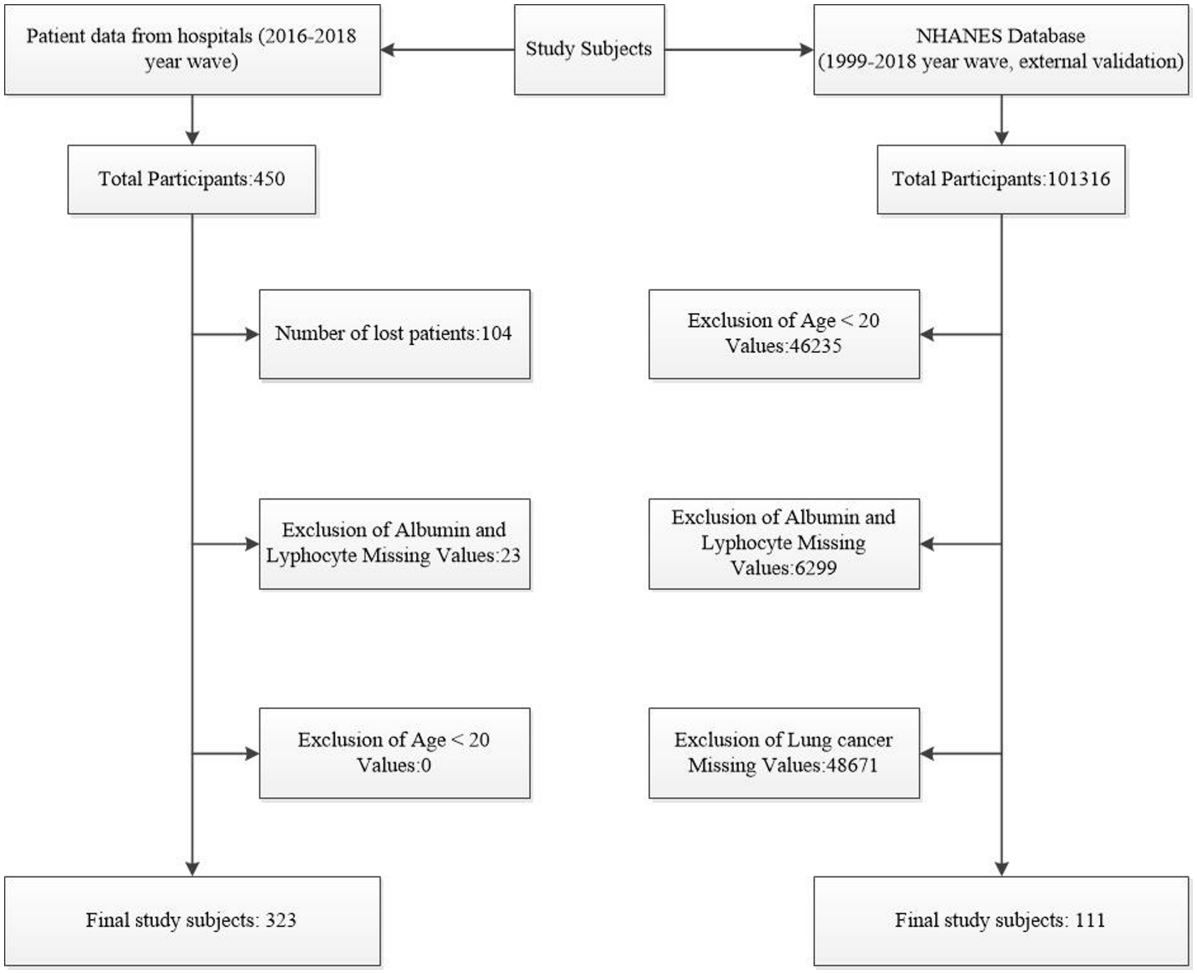
Flowchart of Subject Selection. This figure illustrates the process of selecting subjects from hospital data and the NHANES database.

This hospital-based cohort study included lung cancer patients diagnosed between the 2016–2018 year wave. Inclusion criteria were patients aged ≥20 years with available data on PNI and its components (albumin and lymphocyte count). Exclusion criteria included missing data for albumin, lymphocyte count, or other key variables. For external validation, data from the NHANES database (1999–2018 year wave) were used. The initial study cohort comprised 450 patients, with 104 lost to follow-up and 23 excluded due to missing albumin or lymphocyte data, resulting in 323 subjects for the final analysis. The NHANES database included 10,1,316 individuals, with 111 lung cancer patients selected after excluding 46,235 participants with age < 20, 48,671 missing lung cancer diagnoses and 6,299 missing albumin or lymphocyte values (Figure 1).

### 2.2 Variables and definitions

The primary outcome was all-cause mortality. The main exposure was the PNI, calculated as 10 × albumin (g/L) + 0.005 × lymphocyte count (/mm^3^). Variables assessed included age, sex, smoking status, alcohol consumption, hypertension, fasting blood glucose, liver function tests (ALT, AST), renal function tests (serum creatinine, blood urea nitrogen), lipid profiles (triglyceride, total cholesterol, HDL, LDL), and hematological parameters (WBC, lobulated neutrophils, lymphocyte number, RBC distribution width, PLT, Hb).

### 2.3 Cox proportional hazards regression analysis

Univariate Cox regression was first performed to identify factors associated with mortality in lung cancer patients (19). This step helped to screen out potentially relevant variables for further analysis. Next, variance inflation factor (VIF) testing was conducted to detect multicollinearity among the variables. A VIF value greater than 10 typically indicates significant multicollinearity; however, in this study, all VIF values were below 5, suggesting that multicollinearity was not a major issue (20, 21). Subsequently, multivariate Cox regression was carried out to control for confounding variables and determine the independent association of the PNI and its components (albumin and lymphocyte count) with mortality (22). The results were presented as hazard ratios (HRs) with 95% confidence intervals (CIs).

### 2.4 Survival analysis and non-linear relationship assessment

The KM survival curves were plotted to visualize the survival differences across different levels of PNI, albumin, and lymphocyte count. The log-rank test was used to assess the significance of these differences. To further explore the relationship between continuous variables and mortality risk, RCS models were applied. These models allowed us to assess potential non-linear relationships and provided a more comprehensive understanding of how PNI and its components might influence mortality risk (23).

### 2.5 Two-piecewise linear regression analysis

To evaluate the relationship between the Prognostic Nutritional Index (PNI) and lung cancer mortality, we employed a two-piecewise linear regression model. This model allows for the identification of potential threshold effects by examining changes in the slope of the relationship at a specific inflection point. The model was specified to include two segments: one for PNI values below the inflection point and another for values above it. The inflection point was determined using a likelihood ratio test to optimize model fit. This approach helps to identify whether a specific PNI value significantly alters the relationship with the outcome.

### 2.6 Statistical analysis

Measured variables were presented as the mean (standard deviation) or median (tertiles), and count variables were presented as frequencies (percentages). The Kolmogorov–Smirnov test was used to assess the normality of variables. For normally distributed data, intergroup differences were analyzed using *t*-tests or analysis of variance. For skewed data, the Mann–Whitney *U* test or Kruskal–Wallis *H* test was used for intergroup comparisons. Chi-square tests or Fisher’s exact tests were used for count data. PNI was treated as a continuous variable and divided into tertiles in the analysis to minimize the impact of its distribution on the results. Kaplan–Meier (KM) curves (with the log-rank test) were used to evaluate the effect of baseline PNI categories on all-cause mortality. Univariate and multivariate Cox proportional hazards models were used to estimate hazard ratios and 95% confidence intervals, and restricted cubic spline models were employed to assess potential non-linear effects. The ‘segmented’ package in *R* was utilized to conduct a two-piecewise linear regression analysis, identifying the inflection point in the relationship between PNI and lung cancer mortality. In all analyses, a two-tailed *p*-value of less than 0.05 was considered statistically significant. All data analyses were performed using R version 4.4.2.

## 3 Results

### 3.1 Identifying key prognostic factors

The hospital-based single-factor Cox regression analysis revealed that PNI, albumin, and lymphocyte count were significantly associated with lung cancer mortality (Tables 1, 2). Specifically, PNI had an HR of 0.89 (95% CI: 0.85–0.93, *p* < 0.01), indicating a 10% decrease in mortality risk with each unit increase in PNI. Albumin, with an HR of 0.88 (95% CI: 0.86–0.92, *p* < 0.01), showed that higher albumin levels were linked to lower mortality risks. Lymphocyte count, with an HR of 0.60 (95% CI: 0.50–0.80, *p* < 0.01), suggested that patients with higher lymphocyte counts had significantly reduced mortality risks. These findings point to PNI and its components as crucial prognostic factors for lung cancer patients.

**Table 1:** Univariate Cox analysis of hospital patients and external validation via NHANES database.

Characters	Patient data from hospitals		NHANES (external validation)	
	Statistics	MORSTAT	Statistics	MORTSTAT
Sex				
No	113 (35.0%)	1.0	47 (41.8%)	1.0
Yes	210 (65.0%)	1.16 (0.84, 1.61) 0.36	64 (58.2%)	1.39 (0.80, 2.41) 0.24
Age	65.7 ± 9.2	1.02 (1.00, 1.04) 0.01	68.2 ± 11.3	1.07 (1.04, 1.11) < 0.01
Hypertension				
No	228 (70.6%)	1.0	45 (40.0%)	1.0
Yes	95 (29.4%)	1.07 (0.77, 1.48) 0.70	66 (60.0%)	1.25 (0.72, 2.18) 0.43
Smoke				
No	176 (54.5%)	1.0	91 (81.8%)	1.0
Yes	147 (45.5%)	1.20 (0.89, 1.63) 0.23	20 (18.2%)	1.02 (0.48, 2.16) 0.96
Alcohol				
No	250 (77.4%)	1.0	51 (45.5%)	1.0
Yes	73 (22.6%)	1.05 (0.74, 1.51) 0.78	60 (54.5%)	1.03 (0.60, 1.75) 0.92
Fasting blood glucose	5.4 ± 1.8	0.90 (0.82, 0.99) 0.02	132.1 ± 51.3	1.00 (1.00, 1.01) 0.50
ALT	23.4 ± 20.0	0.99 (0.98, 1.00) 0.03	24.1 ± 28.9	0.99 (0.97, 1.01) 0.33
AST	23.0 ± 16.1	1.00 (0.99, 1.01) 0.34	27.9 ± 21.6	1.00 (0.98, 1.01) 0.72
Serum creatinine	71.5 ± 15.1	1.00 (0.99, 1.01) 0.99	1.00 ± 0.3	3.05 (1.33, 7.02) 0.01
Blood urea nitrogen	5.5 ± 1.7	1.03 (0.94, 1.12) 0.51	16.2 ± 7.4	1.03 (1.00, 1.07) 0.08
Triglyceride	1.7 ± 5.0	0.75 (0.58, 0.96) 0.02	122.4 ± 66.9	1.00 (1.00, 1.00) 0.65
Total cholesterol	4.0 ± 0.9	0.71 (0.59, 0.85) < 0.01	188.7 ± 42.6	1.00 (1.00, 1.01) 0.70
HDL	1.1 ± 0.3	0.56 (0.32, 0.95) 0.03	55.9 ± 20.7	1.02 (1.01, 1.03) < 0.01
LDL	3.3 ± 14.5	0.71 (0.58, 0.86) < 0.01	101.00 ± 45.4	1.00 (1.00, 1.01) 0.61
WBC	6.9 ± 2.5	0.99 (0.93, 1.05) 0.70	7.4 ± 2.2	1.03 (0.91, 1.16) 0.64
Lobulated neutrophils	4.6 ± 2.3	1.02 (0.95, 1.09) 0.65	4.7 ± 1.9	1.18 (1.04, 1.35) 0.014
Hb	138.0 ± 20.1	0.99 (0.98, 1.00) < 0.01	13.8 ± 1.4	1.15 (0.97, 1.38) 0.11
RBC distribution width	45.1 ± 4.8	1.03 (1.00, 1.06) 0.09	253.6 ± 71.5	1.00 (1.00, 1.00) 0.97
PLT	218.6 ± 79.1	1.00 (1.00, 1.00) 0.43	1.8 ± 0.9	0.60 (0.40, 0.90) 0.01
Lymphocyte number	1.6 ± 0.6	0.60 (0.50, 0.80) < 0.01	41.1 ± 3.9	0.62 (0.44, 0.87) 0.01
Albumin	41.9 ± 5.5	0.88 (0.86, 0.92) < 0.01	42.9 ± 4.2	0.94 (0.90, 0.98) 0.02
PNI	49.9 ± 6.6	0.89 (0.85, 0.93) < 0.01	42.8 ± 4.1	0.93 (0.87, 0.99) 0.02

This table presents the baseline characteristics of lung cancer patients and their association with mortality, including variables like gender, age, hypertension, smoking, alcohol consumption, fasting blood glucose, liver function, renal function, lipid profiles, and hematological parameters, along with hazard ratios (HR).

**Table 2:** Baseline characteristics of hospital patients and external validation via NHANES database.

MORTSTAT	Patient data from hospitals		NHANES (external validation)	
	No	Yes	No	Yes
*N*	152	171	56	55
Age	64.4 ± 7.9	66.9 ± 10.0	64.4 ± 11.8	72.1 ± 9.4
BMI	27.9 ± 7.1	26.9 ± 7.0	27.9 ± 7.1	26.9 ± 7.0
Fast glucose	5.6 ± 2.1	5.2 ± 1.6	128.0 ± 47.7	136.2 ± 54.7
ALT	25.7 ± 23.9	21.4 ± 15.5	28.2 ± 39.3	20.0 ± 10.1
AST	23.4 ± 19.7	22.6 ± 12.1	29.7 ± 27.4	26.0 ± 13.7
Creatinine	71.5 ± 14.5	71.4 ± 15.7	1.0 ± 0.3	1.1 ± 0.4
Fast triglyceride	2.1 ± 7.3	1.2 ± 0.7	125.1 ± 67.1	119.7 ± 67.3
Blood urea nitrogen	5.5 ± 1.8	5.5 ± 1.6	15.3 ± 6.1	17.1 ± 8.5
HDL	1.2 ± 0.3	1.1 ± 0.4	51.0 ± 16.7	60.9 ± 23.2
LDL	4.3 ± 21.1	2.3 ± 0.8	100.4 ± 46.0	101.6 ± 45.1
WBC	7.0 ± 2.4	6.8 ± 2.6	7.4 ± 2.0	7.4 ± 2.4
Lobulated neutrophils	4.6 ± 2.1	4.7 ± 2.5	4.4 ± 1.5	5.0 ± 2.1
RBC distribution width	44.3 ± 4.3	45.8 ± 5.1	13.8 ± 1.5	13.8 ± 1.2
PLT	226.2 ± 75.3	211.9 ± 82.0	248.4 ± 63.0	258.8 ± 79.5
Albumin	43.9 ± 4.4	40.2 ± 5.8	41.6 ± 4.3	40.5 ± 3.4
Lymphocyte	1.8 ± 0.6	1.4 ± 0.5	2.0 ± 0.8	1.6 ± 0.8
PNI	52.8 ± 5.0	47.4 ± 6.7	43.6 ± 4.6	42.1 ± 3.6
Sex				
0	57 (37.5%)	56 (32.7%)	26 (47.3%)	20 (36.4%)
1	95 (62.5%)	115 (67.3%)	29 (52.7%)	35 (63.6%)
Hypertension				
0	109 (71.7%)	119 (69.6%)	26 (45.5%)	19 (34.5%)
1	43 (28.3%)	52 (30.4%)	30 (54.5%)	36 (65.5%)
Smoke				
0	91 (59.9%)	85 (49.7%)	43 (78.2%)	47 (85.5%)
1	61 (40.1%)	86 (50.3%)	13 (21.8%)	8 (14.5%)
Alcohol				
0	118 (77.6%)	132 (77.2%)	24 (41.8%)	27 (49.1%)
1	34 (22.4%)	39 (22.8%)	32 (58.2%)	28 (50.9%)

This table compares the baseline characteristics of hospital patients and NHANES database subjects between the non-deceased and deceased groups, covering indicators such as age, BMI, glucose, and liver and kidney function, as well as categorical variables including sex, hypertension, smoking, and alcohol consumption. Data are presented as mean ± standard deviation or frequency (percentage) to analyze differences between groups.

### 3.2 Ensuring analytical reliability

Before the hospital-based multivariate Cox regression analysis, VIF testing was conducted to check for multicollinearity among variables (Table 3). All variables had VIF values below 5, such as age (VIF = 1.1) and TOTAL CHOLESTEROL (VIF = 1.3), indicating no severe multicollinearity. As VIF values above 10 suggest significant multicollinearity, the results here imply that the multivariate analysis outcomes were reliable and valid for evaluating the independent connection between PNI, its components, and mortality.

**Table 3:** Variance inflation factor (VIF) analysis.

Patient data from hospitals	VIF	NHANES (external validation)	VIF
Age	1.1	Lobulated neutrophils	1
Fasting blood glucose	1.1	HDL	1
ALT	1	Serum creatinine	1.1
HB	1.1	Age	1.1
Red blood cell distribution width	1.1	PLT	1.1
Triglyceride	1		
Total cholesterol	1.3		
HDL	1.3		
LDL	1		

This table displays VIF values for variables in hospital patient data and the NHANES database to assess multicollinearity. All VIF values are below 5, indicating no severe multicollinearity.

### 3.3 Independent prognostic value of PNI

In the hospital-based multivariable Cox regression analysis model (Adjustment Model I), PNI was independently associated with lung cancer mortality (Table 4). Compared to the low group, the hazard ratio (HR) for the medium group was 0.75 (95% CI: 0.40–1.45, *p* = 0.13), and for the high group was 0.30 (95% CI: 0.10–0.55, *p* = 0.03), indicating a significant reduction in mortality risk with increasing PNI levels. Additionally, in this model, compared to the low group, patients with high lymphocyte counts had an HR of 0.92 (95% CI: 0.48–2.45, *p* = 0.88), and those with high albumin levels had an HR of 0.90 (95% CI: 0.30–2.45, *p* = 0.80). These results confirm the important independent prognostic value of PNI and its components, albumin and lymphocyte count, in predicting lung cancer mortality. This suggests that clinicians should pay attention to these indicators when assessing patient prognoses.

**Table 4:** Association between PNI, lymphocyte count, albumin and mortality.

Exposure	Patient data from hospitals		NHANES (external validation)	
	Crude	Adjust I	Crude	Adjust I
PNI				
Low	1.00	1.00	1.00	1.00
Middle	0.60 (0.35, 1.10) 0.12	0.75 (0.40, 1.45) 0.13	1.10 (0.40, 3.30) 0.84	0.90 (0.50, 5.40) 0.13
High	0.35 (0.10, 0.95) 0.03	0.30 (0.10, 0.55) 0.03	1.30 (0.25, 7.30) 0.79	0.70 (0.10, 0.90) 0.01
Lymphocyte number				
Low	1.00	1.00	1.00	1.00
Middle	1.20 (0.70, 2.00) 0.55	1.10 (0.65, 1.90) 0.78	0.75 (0.35, 1.20) 0.18	0.60 (0.30, 1.20) 0.15
High	1.05 (0.45, 2.50) 0.99	0.92 (0.48, 2.45) 0.88	0.40 (0.20, 0.90) 0.02	0.42 (0.26, 0.90) 0.03
Albumin				
Low	1.00	1.00	1.00	1.00
Middle	0.70 (0.40, 0.85) 0.02	1.00 (0.50, 1.70) 0.92	0.85 (0.30, 2.45) 0.68	0.85 (0.35, 2.80) 0.78
High	0.65 (0.25, 0.90) 0.02	0.90 (0.30, 2.45) 0.80	0.50 (0.10, 0.85) 0.04	0.94 (0.18, 5.60) 0.89

This table shows Cox regression analysis results from hospital data and the NHANES database, highlighting the association of PNI, lymphocyte count, and albumin with all-cause mortality in lung cancer patients in both crude and adjusted models.

### 3.4 Survival differences revealed by Kaplan–Meier curves

**Figure 2:**
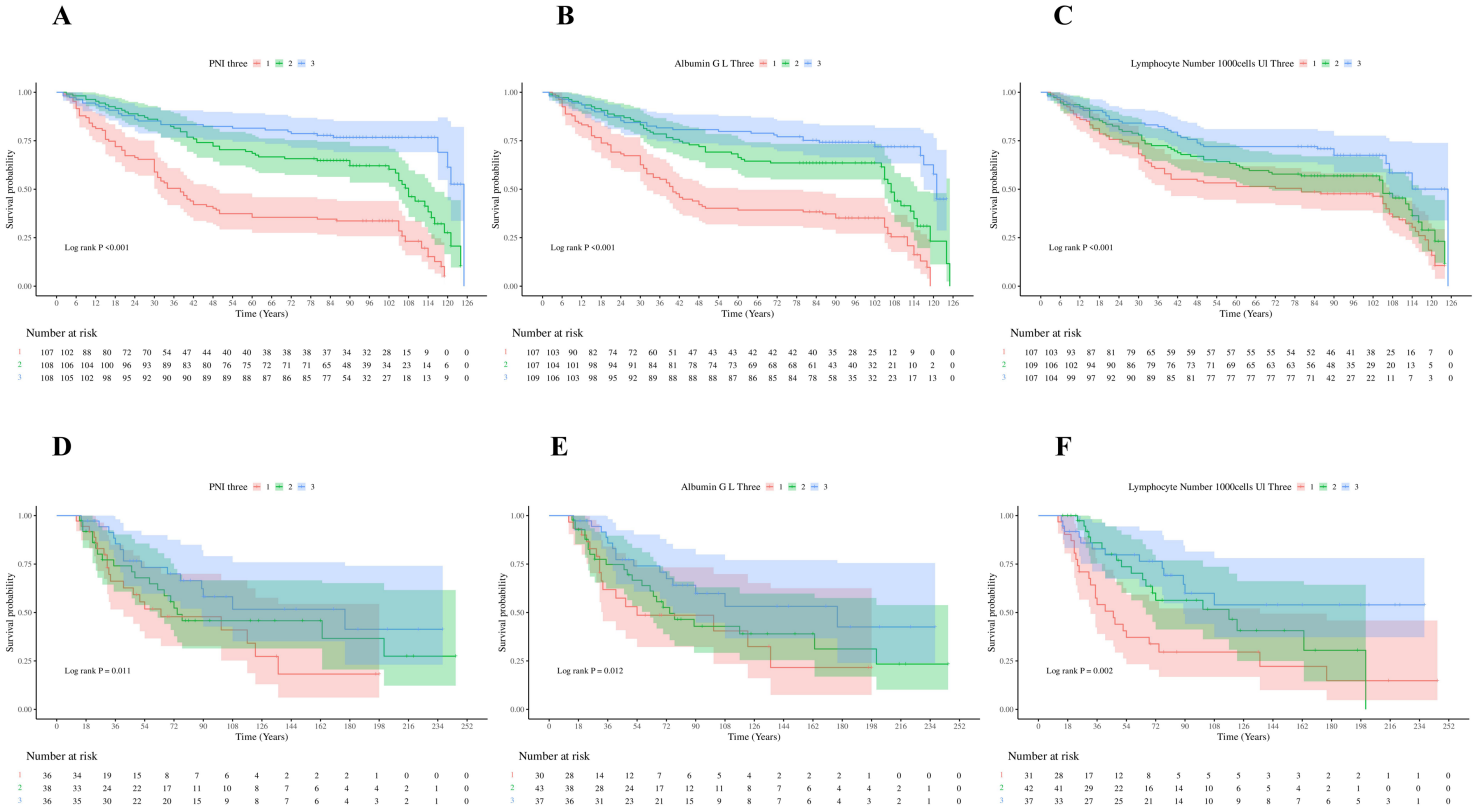
Kaplan–Meier survival curves for PNI, albumin, and lymphocyte count in lung cancer patients. Panels **(A–C)** display the Kaplan–Meier survival curves for PNI, albumin, and lymphocyte count based on hospital data, showing survival differences across different levels (PNI: log-rank *p* < 0.001; Albumin: log-rank *p* < 0.001; Lymphocyte count: log-rank *p* < 0.001). Panels **(D–F)** present the Kaplan–Meier survival curves for these factors based on the NHANES database (PNI: log-rank *p* = 0.011; Albumin: log-rank *p* = 0.012; Lymphocyte count: log-rank *p* = 0.002).

The Kaplan–Meier survival curves based on hospital data clearly showed survival rate differences among patients with different levels of PNI, albumin, and lymphocyte count (Figures 2A–C). In the PNI analysis, the high-PNI group had a significantly higher survival rate than the medium- and low-PNI groups (log-rank *p* < 0.001), indicating better survival outcomes with higher PNI levels. Similarly, patients with high albumin levels had a higher survival rate than those with low levels (log-rank *p* < 0.001), highlighting albumin’s positive impact on long-term survival as a nutritional indicator. Patients with high lymphocyte counts also had a higher survival rate (log-rank *p* < 0.001), likely due to lymphocytes’ key role in immune defense and tumor surveillance. These significant survival differences validate the predictive value of these indicators for lung cancer patient prognoses and suggest that monitoring them can aid in risk stratification and individualized treatment decisions.

### 3.5 Exploring non-linear relationships

**Figure 3:**
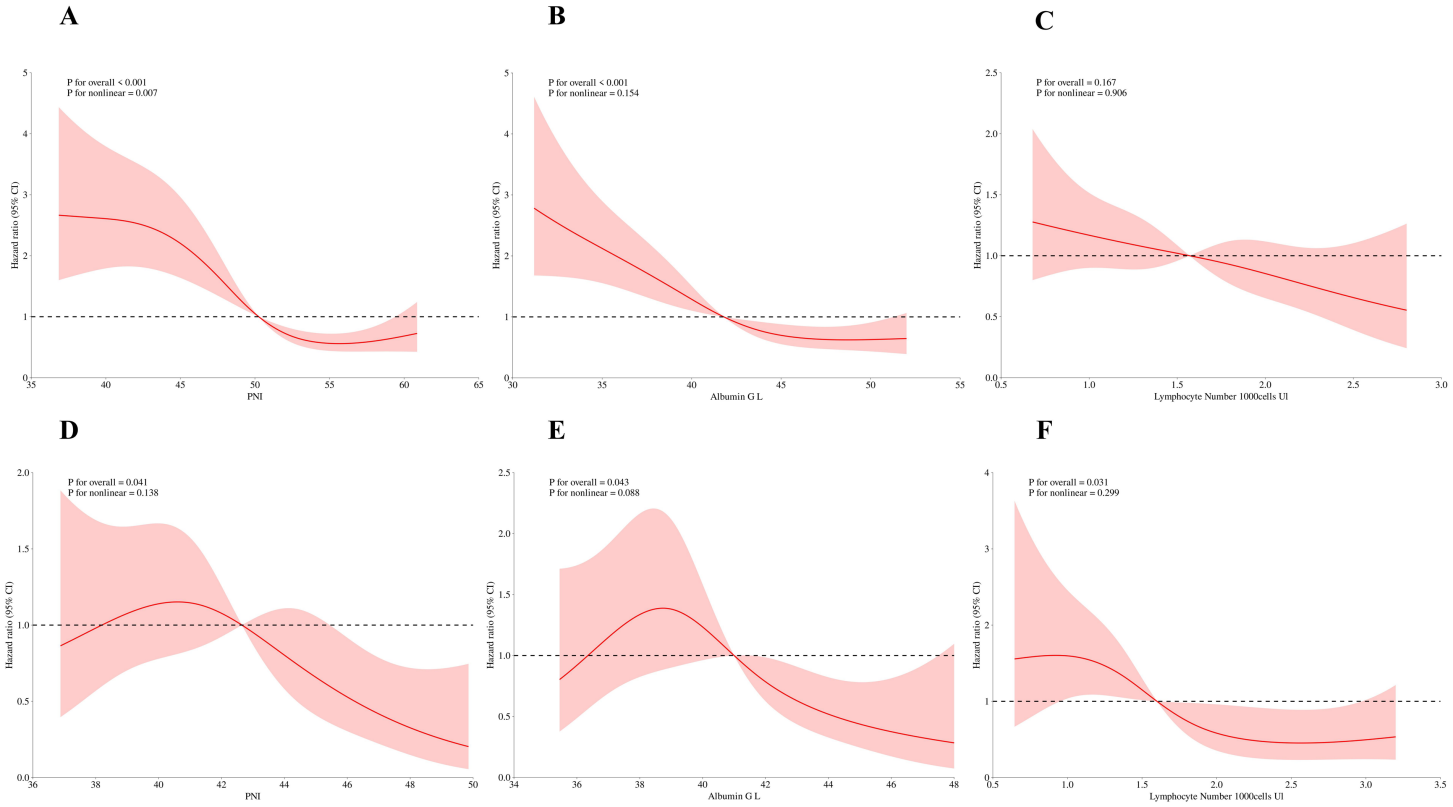
Non-linear relationships between PNI, albumin, lymphocyte count and all-cause mortality. Panels **(A–C)** show the non-linear relationships between PNI, albumin, lymphocyte count and all-cause mortality based on hospital data (PNI: *P* for overall < 0.001, *P* for non-linear = 0.007; Albumin: *P* for overall < 0.001, *P* for non-linear = 0.154; Lymphocyte count: *P* for overall = 0.167, *P* for non-linear = 0.906). Panels **(D–F)** show these relationships based on the NHANES database (PNI: *P* for overall = 0.041, *P* for non-linear = 0.138; Albumin: *P* for overall = 0.043, *P* for non-linear = 0.088; Lymphocyte count: *P* for overall = 0.031, *P* for non-linear = 0.299).

The RCS analysis delved into the potential non-linear relationships between PNI, albumin, lymphocyte count, and mortality risk (Figures 3A–C). The analysis showed a significant non-linear relationship between PNI and mortality risk (*P* for overall <0.001, *P* for non-linear = 0.007), suggesting that PNI increases were associated with mortality risk decreases within a certain range, but the relationship wasn’t strictly linear. There might be an optimal PNI range for the best patient survival outlook. Similarly, a significant non-linear relationship was found between albumin and mortality risk (*P* for overall <0.001, *P* for non-linear = 0.154), indicating that albumin levels might have a threshold effect on mortality risk. However, no significant non-linear relationship was found between lymphocyte count and mortality risk (*P* for overall = 0.167, *P* for non-linear = 0.906). This could be due to sample size, variation in lymphocyte counts, or other confounding factors. Overall, the RCS analysis indicates that the effects of PNI and albumin on mortality might be more complex and require further research to clarify their dose-response relationships.

### 3.6 Confirming the effectiveness of predictive indicators through external validation

External validation results confirmed the significant association between PNI and mortality, aligning with the hospital-based findings (Tables 1–4 and Figures 2D–F). The Kaplan–Meier survival curve analysis showed significant survival rate differences among patients with different PNI levels in the NHANES database (log-rank *p* = 0.011), echoing the hospital-based results. This indicates that PNI has good predictive power across different populations. In the multivariate Cox regression analysis, the high-PNI group showed a significantly reduced mortality risk (HR = 0.70, 95% CI: 0.10–0.90, *p* = 0.01) compared to the low group. This underscores PNI’s predictive value in the external validation dataset. RCS analysis of the NHANES data indicated no significant non-linear relationships between PNI, albumin, lymphocyte count, and mortality risk (*P* for overall were 0.041, 0.043, and 0.031, respectively; *P* for non-linear were 0.138, 0.088, and 0.299, Figures 3D–F). This suggests a more linear relationship between these indicators and mortality risk in the external validation data, differing from the non-linear relationships in the hospital-based data. Overall, external validation results strongly support the validity of PNI and its components as mortality-risk predictive indicators. However, their performance varies across populations, indicating a need for further research to optimize PNI application strategies and enhance its predictive accuracy across different groups.

### 3.7 Threshold effects of PNI on lung cancer mortality

Table 5 shows that the relationship between PNI and lung cancer mortality has a significant threshold effect. In the NHANES external validation data, the inflection point of PNI is 42.70. When PNI is below 42.70, there is no significant association between PNI and lung cancer mortality (HR = 1.08, 95% CI: 0.89–1.30, *p* = 0.43). However, when PNI is greater than or equal to 42.70, PNI is significantly negatively correlated with lung cancer mortality (HR = 0.79, 95% CI: 0.64–0.99, *p* = 0.04). In the hospital patient data, the inflection point of PNI is 55.95. When PNI is below 55.95, PNI is significantly negatively correlated with lung cancer mortality (HR = 0.90, 95% CI: 0.88–0.93, *p* < 0.001). However, when PNI is greater than or equal to 55.95, there is no significant association between PNI and lung cancer mortality (HR = 1.05, 95% CI: 0.89–1.23, *p* = 0.59). These results indicate that the impact of PNI on lung cancer mortality varies significantly at different levels, and the inflection points also differ between data sources. This suggests that in clinical practice, the threshold of PNI should be determined based on specific data sources and patient groups to more accurately assess the prognosis of lung cancer patients.

**Table 5:** Inflection point analysis of PNI and its impact on lung cancer mortality.

Data source	Outcome	Effect	*P*
NHANES (external validation)	Inflection point	42.70	
	<42.70	1.08 (0.89–1.30)	0.43
	≥42.70	0.79 (0.64–0.99)	0.04
	*P* for likelihood test		0.043
Patient data from hospitals	Inflection point	55.95	
	<55.95	0.90 (0.88–0.93)	<0.001
	≥55.95	1.05 (0.89–1.23)	0.59
	*P* for likelihood test		0.038

This table presents the inflection point analysis of PNI from NHANES external validation and hospital patient data. The inflection point indicates the PNI value at which the effect on the outcome significantly changes. The table lists the effect and 95% confidence intervals for PNI values below and above the inflection point, as well as the p-values for the likelihood ratio test to assess model fit.

## 4 Discussion

Our study robustly establishes the PNI as a significant predictor of all-cause mortality in lung cancer patients, with consistent results across various analytical methods and datasets. This consistency strengthens the validity of PNI as a prognostic tool. PNI captures both nutritional and immune statuses, which are crucial for cancer progression and treatment outcomes (24).

The biological basis of PNI’s effectiveness lies in the interplay between nutrition and immunity (25). Serum albumin, reflecting nutritional status, is vital for immune function and metabolic support. Adequate nutrition enhances immune responses, while malnutrition and hypoalbuminemia weaken them, increasing infection and treatment complication risks (26, 27). Lymphocytes, key players in adaptive immunity, mediate immune responses against cancer. A low lymphocyte count indicates impaired immune surveillance, facilitating tumor growth (28). By integrating albumin and lymphocyte count, PNI provides a comprehensive assessment of a patient’s physiological state.

Numerous studies support our findings. For instance, a multicenter retrospective study by Fan et al. found that immune-nutritional parameters, including PNI, significantly predict postoperative complications and mortality in elderly lung cancer patients (29). Another study demonstrated that preoperative PNI is an independent predictor of surgical prognosis in hepatocellular carcinoma patients undergoing open hepatectomy (30). These studies reinforce PNI’s validity as a prognostic indicator across different cancer types. Furthermore, to gain a deeper understanding of the clinical utility of PNI, we compared it with other well-established prognostic scoring systems, such as the Glasgow Prognostic Score (GPS) and the Controlling Nutritional Status (CONUT) score. In contrast to GPS, which is more sensitive in assessing the inflammatory status, PNI, by incorporating lymphocyte count, may offer an advantage in predicting responses to immunotherapy (31). Compared to the CONUT score, PNI’s streamlined scoring system could be more readily implemented in clinical practice, potentially reducing variability due to laboratory testing errors (32). This comparison underscores the potential of PNI as a valuable tool in the prognostic assessment of lung cancer patients.

The non-linear relationship between PNI and mortality risk observed in our hospital-based data suggests an optimal PNI range for the best survival outcomes. This may reflect the complex balance between nutritional support and immune activation. Extremely high or low albumin or lymphocyte levels may disrupt this balance, leading to suboptimal outcomes. In contrast, the more linear relationship in the NHANES data indicates that population-specific factors can influence the PNI-mortality association. Furthermore, we have noted that the relationship between PNI and mortality risk exhibits non-linear characteristics in the hospital-based cohort, while it appears more linear in the NHANES cohort. This difference may be associated with the threshold effects of inflammation, the regulatory role of nutritional status, and physiological response variations among different populations. Specifically, the interplay between inflammation and nutrition may influence mortality risk in a non-linear fashion at different PNI levels, and genetic and environmental factors may further modulate this relationship (33). Additionally, a higher proportion of severely ill patients in the hospital cohort may contribute to the stronger non-linear association observed. To better understand these differences, future research is needed across a broader and more diverse range of populations to optimize the application of PNI across various demographics.

In clinical practice, PNI could facilitate individualized treatment strategies. Identifying patients with low PNI values enables early interventions, such as nutritional support or immunotherapy, potentially improving survival outcomes (34). For example, a study by Xia et al. (35) showed that PNI is a significant predictor of survival in patients with non-small cell lung cancer, highlighting its utility in clinical decision-making. Additionally, monitoring PNI changes during treatment could provide real-time prognostic information, allowing timely treatment adjustments (36).

In addition, our study highlights PNI as a significant predictor of all-cause mortality in lung cancer patients, offering a comprehensive assessment of their nutritional and immune statuses. The potential of PNI extends beyond mere prediction; it can also guide the development of personalized treatment plans, including early interventions and decisions regarding immunotherapy. Integrating PNI into electronic health records can enhance evidence-based patient management. However, integrating PNI into clinical workflows presents several challenges. Firstly, a cost–benefit analysis is necessary to evaluate the relationship between the costs of PNI testing and the potential health benefits derived from improved prognostic assessments. Secondly, establishing optimal PNI cutoff values is crucial for ensuring accuracy across diverse populations. Additionally, comorbidities such as infections or chronic liver diseases may affect the components of PNI, thereby impacting its predictive accuracy. Lastly, integrating PNI into electronic health records and clinical decision support systems may require additional technical support and training. Despite these challenges, through multidisciplinary collaboration and further research, PNI has the potential to be effectively incorporated into clinical workflows, ultimately improving prognostic assessments and treatment decisions for lung cancer patients.

Future research should explore the therapeutic implications of targeting PNI components. Nutritional interventions to boost albumin levels and immunotherapies to enhance lymphocyte counts could be evaluated for their potential to improve patient outcomes. Furthermore, investigating the molecular mechanisms underlying the PNI-mortality relationship may uncover new therapeutic targets. For example, research on the role of albumin in modulating the tumor microenvironment and the specific immune mechanisms by which lymphocytes combat cancer cells could provide deeper insights into cancer biology.

In conclusion, our study provides robust evidence highlighting the association of PNI with all-cause mortality in lung cancer patients. The biological plausibility of PNI, as supported by existing literature, and its demonstrated predictive power across different datasets, suggest that PNI could serve as a valuable tool for risk assessment and potentially inform treatment planning. However, our findings do not establish causality, and further research is needed to explore the underlying mechanisms and the therapeutic implications of targeting PNI components. Future studies should aim to elucidate the causal pathways linking PNI to mortality outcomes and to determine how PNI could be optimally integrated into clinical workflows to enhance patient care.

## 5 Limitations

This study recognizes several limitations. The observed differences between hospital and NHANES data may be attributed to demographic and lifestyle variations, as well as differing clinical environments. The smaller sample size of the NHANES cohort could potentially impact the accuracy of our findings. Furthermore, although our research indicates a significant association between PNI and lung cancer patient mortality, we acknowledge that the components of PNI—albumin and lymphocyte count—may be influenced by factors such as inflammation, liver and kidney function, and acute infections. These factors could act as potential confounders affecting the relationship between PNI and mortality. However, due to the constraints of our study design, we were unable to fully control for all these potential confounding variables. Future research employing more sophisticated statistical methods, such as propensity score matching or multivariate regression models, could better account for these confounders, thereby offering more precise estimates of causal relationships.

## Data Availability

The original contributions presented in the study are included in the article/supplementary material, further inquiries can be directed to the corresponding author.
